# Acute Mitral Regurgitation: An Unusual Cause of Unilateral Pulmonary Consolidation

**DOI:** 10.7759/cureus.12707

**Published:** 2021-01-14

**Authors:** Mohammed Mahdi, Fatemeh Abbasi, Maria Mironova, Manish Gugnani, Pirouz Parang

**Affiliations:** 1 Internal Medicine, Capital Health Regional Medical Center, Trenton, USA; 2 Pulmonary and Critical Care Medicine, Capital Health Regional Medical Center, Trenton, USA; 3 Cardiology, Capital Health Regional Medical Center, Trenton, USA

**Keywords:** unilateral, pulmonary edema, mitral valve insufficiency

## Abstract

Unilateral pulmonary consolidation generally indicates infectious pneumonia. In this case report, we describe a patient with infective endocarditis and acute mitral valve regurgitation who developed acute unilateral pulmonary consolidation that resolved dramatically after mechanical ventilation and diuretic therapy. The prompt resolution of the consolidation with treatment suggests pulmonary edema. This case report highlights that rare conditions such as acute pulmonary edema should be considered in the differential diagnosis of patients who present with unilateral pulmonary consolidation to avoid delay in appropriate treatment.

## Introduction

Pulmonary edema is an emergency where there is an acute alveolar fluid accumulation that impairs ventilation and gas exchange [1]. It can be caused by increased hydrostatic pressure (cardiogenic) or increased permeability of the pulmonary capillaries (non-cardiogenic) [1,2]. While bilateral pulmonary edema can be caused by these two mechanisms, unilateral pulmonary edema is known to be mostly cardiogenic [3]. Acute severe mitral regurgitation is one of the causes of cardiogenic pulmonary edema. The non-dilated (non-remodeled) left atrium transmits the regurgitant jet and its pressure to the pulmonary veins. The right pulmonary veins are the most commonly involved veins leading to unilateral right pulmonary edema [3]. Here, we present a case of pulmonary edema manifesting as unilateral pulmonary consolidation mimicking pneumonia. This article was previously presented as a meeting poster at the 2020 CHEST annual meeting on October 18-21, 2020.

## Case presentation

A 30-year-old African American female was admitted prior to the pandemic of coronavirus disease 2019 for the management of non-ST elevation myocardial infarction. Her active medical history was significant for methicillin-sensitive *Staphylococcus aureus* (MSSA) bacteremia, infective endocarditis, mitral and tricuspid valve vegetations, septic pulmonary embolism, vertebral osteomyelitis, cardiac tamponade status post pericardiotomy, and intravenous drug use. She was receiving intravenous infusion of unfractionated heparin, intravenous broad-spectrum antibiotic therapy with vancomycin and cefepime, aspirin, clopidogrel, metoprolol, and atorvastatin therapy. On the seventh day of hospitalization, she developed sudden-onset acute dyspnea, altered mental status, and low oxygen saturation. Her blood pressure was 109/63 mmHg, heart rate 117 bpm, and oxygen saturation of 79% on 3 L oxygen nasal canula, which increased to 89% on non-rebreather mask. Physical examination was remarkable for grade 3 holosystolic murmur with muffled s1 and s2 without s3, normal jugular venous pressure, and no lower extremity edema or cyanosis. The laboratory findings are summarized in Table 1.

**Table 1:** Laboratory findings on admission and during rapid response evaluation. NT-proBNP, N-terminal pro b-type natriuretic peptide; PaCO_2_, partial pressure of arterial carbon dioxide; PaO_2_, partial pressure of arterial oxygen

Variable	Day one	Day seven	Reference range
White blood count	16,000	22,300	4,000-10,000 (cells/uL)
Hemoglobin	11.3	10.9	11.2-15.7 (g/dL)
Platelets	1,88,000	4,02,000	1,50,000-4,00,000 (cells/uL)
Neutrophils	59%	83%	35-70%
Lymphocyte	3%	9%	20-53%
Bands	35%	9%	0-8%
Total protein	7.4	7.2	6.5-8.5 (g/dL)
Albumin	3.2	2.8	3.5-5.0 (g/dL)
Total bilirubin	0.8	0.6	0.2-1.3 (mg/dL)
Aspartate aminotransferase	74	29	14-36 (U/L)
Alanine transaminase	31	11	0-34 (U/L)
Alkaline phosphatase	125	59	38-126 (U/L)
Sodium	138	136	137-145 (mmol/L)
Potassium	3.1	4.6	3.5-5.1 (mmol/L)
Chloride	97	102	98-107 (mmol/L)
HCO_3_	30	22	22-30 (mmol/L)
Urea	28	14	7-17 (mg/dL)
Creatinine	1.0	0.60	0.52-1.04 (mg/dL)
Calcium	9.2	8.5	8.6-10.3 (mg/dL)
NT-proBNP	NA	10,616	0-449 (pg/mL)
Troponin I	6.574	0.911	0-0.034 (ng/mL)
Arterial pH	NA	7.51	7.35-7.45
PaCO_2_	NA	29	35-45 (mmHg)
PaO_2_	NA	53	80-100 (mmHg)

**Figure 1:**
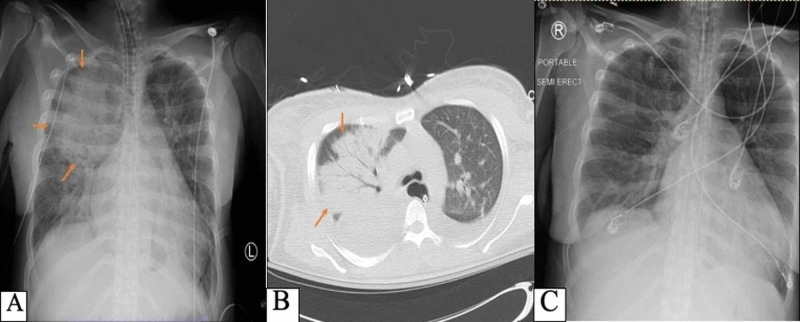
Pre- and post-mechanical ventilation chest imaging. Unilateral right upper lobe consolidation (arrows) evident on anteroposterior chest X-ray (A) and chest CT scan with contrast (B). A follow-up chest X-ray shows its resolution 24 hours after mechanical ventilation and intravenous diuretics (C). CT, computed tomography

It was noted that N-terminal pro b-type natriuretic peptide (NT-proBNP) level had increased to 10,616 from 246 pg/mL four months ago. Anteroposterior view of chest X-ray and chest computed tomography (CT) scan showed new right unilateral pulmonary consolidation (Figure 1).

**Figure 2:**
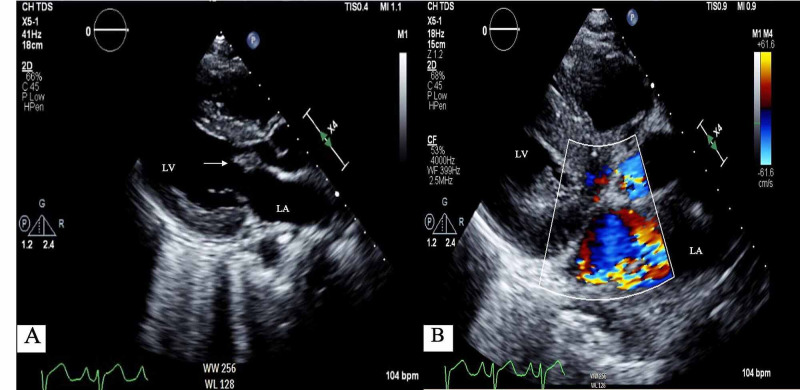
TTE. (A) Parasternal long axis view clearly shows vegetation of the anterior leaflet of mitral valve (arrow) with moderate-to-severe turbulent mitral regurgitation with eccentric jet to LA posterior wally. (B) LV end-diastolic dimension 4.1 cm, LV end-systolic dimension 2.7 cm, and LV ejection fraction (biplane) 68.3%. LA, left artial; LV, left ventricular; TTE, transesophageal echocardiography

Transthoracic echocardiography (TTE) five days earlier showed normal left ventricular ejection fraction and left atrial size, multiple mitral valve vegetations each measuring 1 cm in length, tricuspid valve vegetation having increased in size to 1.9 cm, and moderate-to-severe mitral regurgitation with eccentric posteriorly directed jet (Figure 2).

TEE a month earlier was remarkable for mild mitral regurgitation, moderate tricuspid regurgitation, mitral valve vegetation, and 1.3 cm tricuspid valve vegetation. Magnetic resonance imaging of the brain was remarkable for extensive supratentorial, bilateral, rim-enhancing lesions compatible with septic emboli.

The differential diagnosis at that point included hospital-acquired pneumonia, aspiration pneumonia, septic pulmonary embolism, acute pulmonary edema, and mucous plug. The patient was intubated because of acute hypoxic respiratory failure and transferred to the intensive care unit. Intravenous furosemide 60 mg was initiated immediately and intravenous antibiotics were continued. The next day, as the patient had a dramatic resolution of her right upper lobe consolidation, we did not proceed with bronchoscopy evaluation. She was referred to a tertiary center for mitral valve repair surgery.

## Discussion

Management of cardiogenic pulmonary edema is time-sensitive and centered on three pillars: (i) preload reduction with either diuretics, opiates, nitrates, or ultrafiltration; (ii) afterload reduction with angiotensin-converting enzyme inhibitors, angiotensin receptor blockers, nitroprusside, or intraaortic balloon pump; and (iii) positive inotropic therapy [1].

Unilateral pulmonary consolidation is likely to be diagnosed as pneumonia, especially in the acute setting. A significant cardiac history should warrant measurement of serum BNP and TTE for evidence of structural heart disease, valvular heart disease, or left ventricular dysfunction [2]. Attias et al. reported that unilateral presentation of pulmonary edema is considered to be an independent risk factor for mortality that could be attributed to the delay in appropriate treatment [3].

When uncertain about the diagnosis, advanced tools such as pulmonary artery catheterization can be helpful; however, its use has been associated with increased adverse events without change in overall mortality and hospitalization [4]. TEE is utilized for further evaluation of structural valvular disease that might require surgical intervention [5,6]. While these tools are necessary, they should not delay the treatment initiation. Urgent surgical repair or replacement of mitral valve remains the mainstay therapy of acute mitral valve regurgitation [5,6].

In our case, it is noticeable that the leukocyte count was persistently elevated despite broad-spectrum antibiotic therapy. In fact, the presence of infective endocarditis with systemic embolism can be a possible explanation. Moreover, the confluent and dense-appearing opacities would be a characteristic of alveolar and interstitial fluid, unlike viral pneumonia, which would manifest in patchy and ground glass opacities. Additionally, a quick and complete resolution of pulmonary consolidation after the diuretic therapy and mechanical ventilation does not usually occur in either bacterial or viral pneumonia.

The findings of infective endocarditis with progressively worsening vegetation of mitral valve, classic clinical presentation of acute hypoxic respiratory failure, elevated NT-proBNP, and eccentric mitral regurgitation jet toward the posterior left atrium on TTE favored the diagnosis of cardiogenic pulmonary edema.

## Conclusions

Unilateral pulmonary consolidation should be investigated to rule out pulmonary edema, especially in the presence of known acute cardiac abnormalities, thereby avoiding diagnostic inertia and its consequences of delay in medical management.
